# Systems Biology and Omics Approaches for Complex Human Diseases

**DOI:** 10.3390/biom13071080

**Published:** 2023-07-06

**Authors:** Kumar Selvarajoo, Alessandro Giuliani

**Affiliations:** 1 Bioinformatics Institute (BII), Agency for Science, Technology and Research (A*STAR), Singapore 138671, Singapore; 2 Synthetic Biology for Clinical and Technological Innovation (SynCTI), National University of Singapore (NUS), Singapore 117456, Singapore; 3 School of Biological Sciences, Nanyang Technological University (NTU), Singapore 639798, Singapore; 4 Environment and Health Department, Istituto Superiore di Sanità, Viale Regina Elena 299, 00161 Roma, Italy

For many years, there has been general interest in developing virtual cells or digital twin models [1,2]. This is due to (i) a purely curiosity-driven need to build theories of life and, (ii) the potential to quickly and safely find cures for diseases by testing drugs in silico before testing them on actual organisms. In any case, systems biology, which combines the strengths of physics, chemistry, computer science, mathematics and engineering concepts with the analysis of biological data or phenomena, has been employed in this field. The constraints imposed by the need to use a physically plausible model is a strong barrier against the overfitting and irrelevance of purely computational approaches [3]. This is especially important since large temporal and spatial data that are crucial for complex models are generated on a regular basis [4]. Thus, in this Special Issue, we intend to showcase some of the latest innovative research and views adopting integrative approaches in biology and diseases with an interdisciplinary perspective.

The first paper by Fedeli and colleagues [5] discusses on the theoretical framework that is necessary to understand complex cancers. They review the most popular cancer theory to date, also known as the Somatic Mutation Theory of Carcinogenesis [6], and highlight the controversy of considering only the power of gene mutation as the casual factor for cancers’ origin or onset. They reviewed many works containing recent evidence that cancer can be generated or normal cells can become transformed into cancer cells via a myriad mechanisms at the different omics and network levels [7]. For example, the cancer microenvironment, which constitutes innate and adaptive immune cells, can induce cell proliferation in neighbouring cells [8,9]. Thus, the authors recommend a novel approach to developing a theory that expands on mutation to include cell-to-cell interactions and data from multiple omics studies. This, in their opinion, will solve the current paradoxes observed in cancer biology.

Another paper by Bizzari and co-workers [10] deals with the weaknesses of the Somatic Mutation Theory and remarks that the malignant-to-benign cancer cell transition does not require gene mutation. State transitions can be caused by epigenetic gene expression changes at a network level, without genetic mutation, and many of these state transitions in biology are reversible, e.g., the aerobic to anaerobic states observed in microbes [11]. Here, the authors recommend that models of cancer should take reversibility and non-linearity or bifurcation effects into account. Knowing about and theoretically modelling these effects first will surely allow scientists to phenotypically reprogramme them using novel drugs for safe cancer apoptosis.

Systems biologists or digital twin scientists should curate biological pathways that are implicated in a particular disease and use this information to create virtual models using various methods, such as Boolean, Kinetic or Bayesian statistical models. This is a manual and time-consuming task. Nietert and team [12] created an efficient way to deal with disease maps, which they define as “a systems biological map or model that combines metabolic, signaling, and physiological pathways to create a comprehensive overview of known disease mechanisms.” They used text mining algorithms to analyse scientific publications, which takes readable text passages and convert them into a structured, machine-usable data format. They also developed a user-friendly and interactive disease map viewer that lays text mining results over a systems biology map. This tool, in our opinion, will be very useful for systems biologists working on disease networks.

The dynamical network biomarker (DNB) theory is crucial for predicting an event prior to state transition, such as the onset of disease states [13]. The paper by Haruki et al. [14] uses a Raman spectroscopy method to study activated T cells’ behaviour over time. Unlike traditional methods that use gene expression datasets, here, they are able to track live cells and tissues with detailed molecular fingerprints using label-free non-invasive imaging. After 48 h of tracking, they were able to detect an early T cell transition state signal at 6 h that had not been previously known.

Rabbany and colleagues [15] described a systems biology approach to identify and characterise functional responses and molecular regulatory networks when endothelial cells are subjected to cyclic stretch. Their transcriptomics data analytics identified four key responses: cell cycle regulation, the inflammatory response, fatty acid metabolism, and mTOR signalling, which are driven by eight transcription factors. The study sheds important light onto atheroprotective-to-atheroprone state transitions, which, according to them, can have positive implications for future vascular disease treatments.

The final paper by Zhou and co-workers proposes a Baysian method to studying Skewed X chromosome inactivation (XCI-S), which occurs in certain diseases [16]. Although several computational methods exist, the authors highlighted that estimating 𝛾 based on general pedigrees is a huge challenge. Thus, they thoroughly studied the truncated normal prior and the uniform prior distributions for 𝛾. By applying decomposition methods (eigenvalue and Cholesky), they reduced the computational time. They also rigorously compared their model with the outcomes from two other methods and precisely and accurately achieved the interval estimation of 𝛾. 

Overall, we believe these Special Issue articles add value to systems biology and digital twin research, and I hope that the readers will enjoy and cite these works in the future. Finally, as Albert Einstein said, “everything should be made as simple as possible, but not simpler”; we also feel that the theories and models developed for biology should also follow a similar rule.
